# Impaired Functional Criticality of Human Brain during Alzheimer’s Disease Progression

**DOI:** 10.1038/s41598-018-19674-7

**Published:** 2018-01-22

**Authors:** Lili Jiang, Danyang Sui, Kaini Qiao, Hao-Ming Dong, Luonan Chen, Ying Han

**Affiliations:** 1 CAS Key Laboratory of Behavioral Science, Institute of Psychology, Beijing, 100101 China; 2 Lifespan Connectomics and Behavior Team, Institute of Psychology, Chinese Academy of Sciences, Beijing, 100101 China; 3 Princeton Neuroscience Institute, Princeton University, Princeton, NJ 08544 USA; 4 Department of Psychology, University of Chinese Academy of Sciences, Shijingshan, Beijing, 100049 China; 5 Key Laboratory of Systems Biology, Innovation Center for Cell Signaling Network, Institute of Biochemistry and Cell Biology, Shanghai Institutes for Biological Sciences, Chinese Academy of Sciences, 320 Yue Yang Road, Shanghai, 200031 China; 6 Department of Neurology, XuanWu Hospital of Capital Medical University, Beijing, 100053 China; 7 Center of Alzheimer’s Disease, Beijing; Institute for Brain Disorders, Beijing, 100053 China; 8 Beijing Institute of Geriatrics, Beijing, 100053 China; 9 National Clinical Research Center for Geriatric Disorders, Beijing, 100053 China; 10 PKU Care Rehabilitation Hospital, Beijing, 100053 China

## Abstract

The progression of Alzheimer’s Disease (AD) has been proposed to comprise three stages, subjective cognitive decline (SCD), mild cognitive impairment (MCI), and AD. Was brain dynamics across the three stages smooth? Was there a critical transition? How could we characterize and study functional criticality of human brain? Based on dynamical characteristics of critical transition from nonlinear dynamics, we proposed a vertex-wise Index of Functional Criticality (vIFC) of fMRI time series in this study. Using 42 SCD, 67 amnestic MCI (aMCI), 34 AD patients as well as their age-, sex-, years of education-matched 54 NC, our new method vIFC successfully detected significant patient-normal differences for SCD and aMCI, as well as significant negative correlates of vIFC in the right middle temporal gyrus with total scores of Montreal Cognitive Assessment (MoCA) in SCD. In comparison, standard deviation of fMRI time series only detected significant differences between AD patients and normal controls. As an index of functional criticality of human brain derived from nonlinear dynamics, vIFC could serve as a sensitive neuroimaging marker for future studies; considering much more vIFC impairments in aMCI compared to SCD and AD, our study indicated aMCI as a critical stage across AD progression.

## Introduction

Alzheimer’s Disease (AD) is a chronic neurodegenerative disease characterized by a decline in cognitive and memory functions. It is estimated that 5.2 million Americans have AD and someone in America develops AD every 67 seconds^1^. Not only as increasing proportion of death reason each year, the disease also causes huge family and society burden all over the world. Accumulated evidence demonstrated that the development of AD was slow and progressive over several years to decades, mainly comprising three stages: the preclinical stage subjective cognitive decline (SCD) without impairment in cognition on standard assessments and biomarker evidence for AD, mild cognitive impairment (MCI) with impairment on memory or other domains of cognition on a standard assessment and biomarker evidence for AD, and dementia due to AD with dementia and biomarker evidence for AD^2^. Research on SCD was still on the very early stage, and the difference between SCD and normal state was still rarely reported^3^. Some people with MCI seem to remain stable or return to normal over time, but more than half progress to dementia within 5 years^4^. However, the quantitative mechanism of AD onset and progression was still not clear.

There have been two qualitative descriptions on the dynamical course of AD pathology^5,6^. Jessen *et al*.^2^ proposed a dynamical model of cognitive decline in relation to progressive disease pathology in AD. They hypothesized that, SCD shows subtle decline in cognitive performance but still within the normal cognitive performance range; once cognitive performance declined further and crossed a threshold, the patient entered MCI-AD stage and the cognitive performance begins declining linearly. Meanwhile, Jack *et al.*^5^ proposed a hypothetical dynamical biomarker model of pathophysiological process in AD. They believed that different biomarkers exhibited different sigmoid time course: cerebrospinal fluid (CSF) amyloid beta (Aβ) 42 firstly accumulate, then amyloid Positron Emission Tomography (PET) emerged, then CSF tau proteins starts to accumulate, then brain structure changes (observed using Magnetic Resonance Imaging, MRI), and then fluorodeoxyglucose (FDG) PET appeared, and finally cognitive impairment was detected. The above two dynamical models of AD progression were both rooted in empirical observations from different dimensions including biochemical, neuroimaging and behavior. Compared with the two integrated theory of AD progression, a singlet brain dynamical model with theoretical basis seems more specific and efficient for targeting the prevention and early warning of AD.

Was there a quantitative dynamical trajectory of AD progression from the level of brain dynamics? Was it smooth or abrupt? Was there critical transition during progression of AD? How could we characterize and study functional criticality of human brain? Could the differences between patients and normal controls reflect dynamical changes across the three stages of AD progression? There was no study addressing these questions now. Generally, human brain dynamics can be modeled as a nonlinear system, and critical transition theory in nonlinear dynamics could help this. The dynamical network biomarkers (DNBs) method^7–9^ was just derived from nonlinear dynamics. It supplied us a great opportunity to characterize and study functional criticality of human brain. Theoretically, DNBs^7^ are a group of measurements or variables (molecules, proteins, genes, voxels, vertices, etc.) with average Pearson’s correlation coefficients across time (PCCs) that drastically increase in absolute values, while the average PCCs of the members of this group with variables outside the group drastically decrease in absolute value, and the average standard deviations (SD) in the group drastically increase whenever the system approaches a critical state. Groups that satisfy these three conditions signal the imminent transition of the system from the current state to another state. Such group of measurements were called DNBs. DNBs have been successfully used for predicting critical transitions of diseases based on biochemical and genomic data, including respiratory disease^10^, depression^11^, episodic migraine^12^ and type 1 diabetes^8^.

Based on local information transfer hypothesis and the above three conditions of DNB, here we proposed a vertex-wise Index of Functional Criticality (vIFC) of resting-state fMRI time series to study functional criticality of human brain during AD progression. Specifically, local information transfer hypothesis meant that spatial neighborhood is more likely to be involved in the DNBs of the system. By calculating vIFC maps of 42 SCD, 67 amnestic MCI (aMCI), 34 AD patients as well as their age-, sex-, years of education-matched 54 normal controls (NC), we aimed to: (1) study functional criticality impairments of human brain for the three different stages of AD progression as well as whether functional criticality of human brain is associated with clinical behavioral measurements; (2) verify which measurement is more sensitive, vIFC or standard deviation, correlations within spatial neighborhood cluster and correlations outside neighborhood cluster of human brain functional networks; (3) demonstrate whether aMCI patients exhibit the most patient-normal differences in vIFC, as well as whether aMCI works as a critical transition across AD progression. Overall, combining fMRI methodologies with nonlinear dynamics, the current vIFC study aimed to supply a sensitive neuroimaging marker for future studies, and would also promote our understanding of the neuroimaging pathology of AD progression from the dynamical perspective.

## Results

### vIFC patterns and its three components in healthy populations

**Figure 1:**
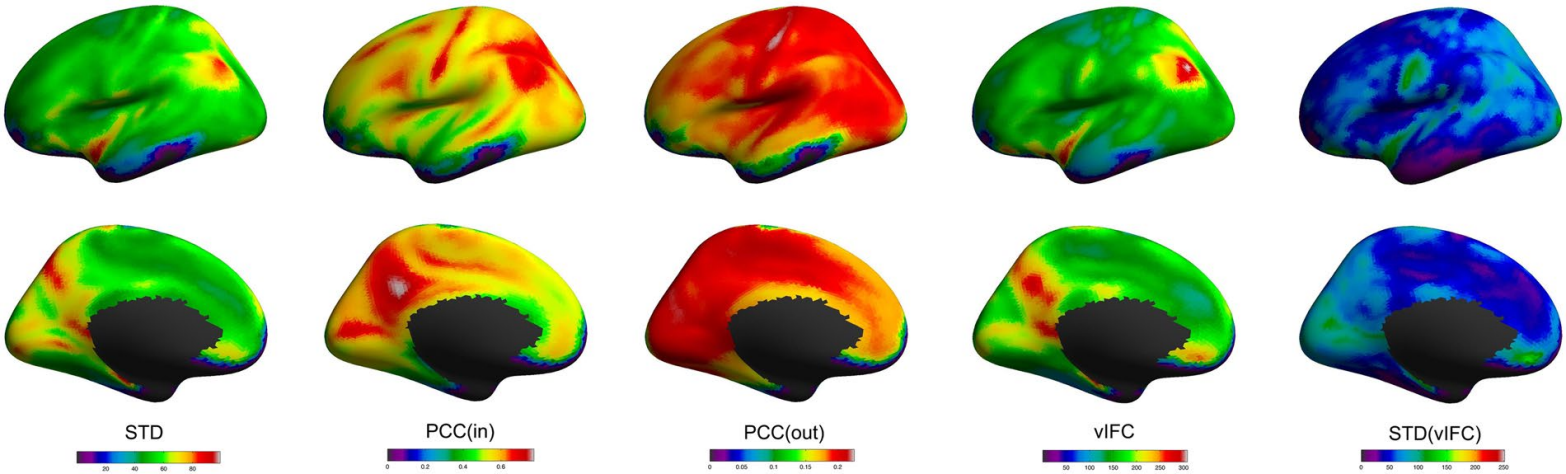
vIFC patterns and its three components in healthy populations. The five columns respectively correspond to the average maps of standard deviation of BOLD time series (STD), within cluster correlation (Corr(in)), outside cluster correlation (Corr(out)), vIFC and individual variability of vIFC (STD(vIFC)) across healthy populations.

To understand how each term of the three components contributes to vIFC, we plotted average maps of standard deviation of BOLD time series (STD), within cluster correlation (Corr(in)) and outside cluster correlation (Corr(out)) together with the average maps of vIFC and individual variability of vIFC (STD(vIFC)) across healthy populations. As shown in Fig. 1, the big STD, Corr(in) and Corr(out) in the inferior parietal and the precuneus contributed the big vIFC in the inferior parietal and the precuneus. For insular, STD and Corr(out) is big but Corr(in) is not very big. In the other hand, the middle/inferior temporal and lateral occipital cortex had small STD, Corr(in) and Corr(out), as well as small vIFC. Standard deviation of vIFC, as a measurement of individual variation, was found to be big mainly in the central sulcus, cuneus, insular, lateral occipital and medial orbitalfrontal cortex, and small in the middle and inferior temporal and medial frontal cortex.

### vIFC alterations across AD progression

**Figure 2:**
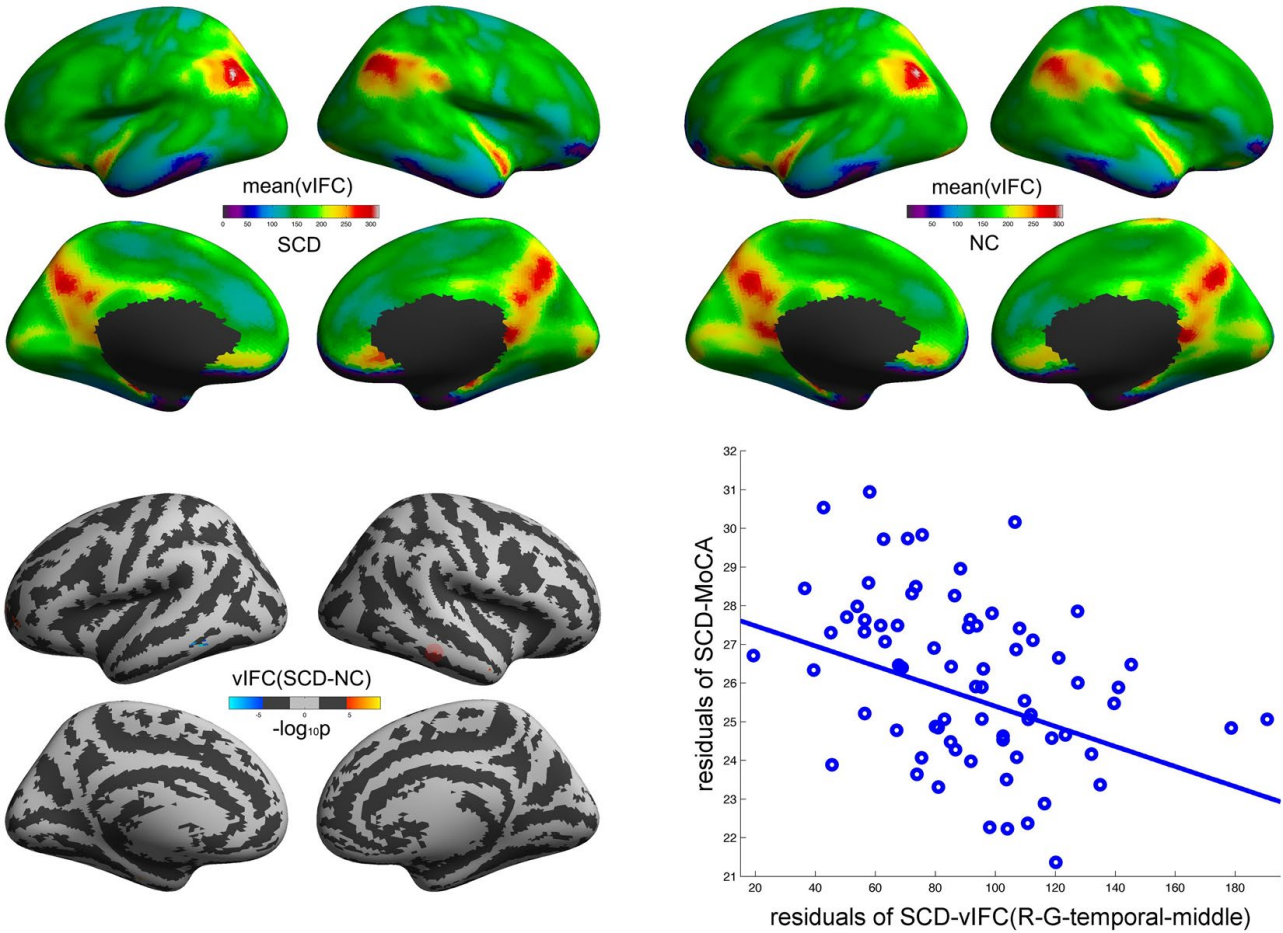
Significant SCD-NC differences in vIFC across cortical mantle. The cold and warm colors respectively stand for significant decreased and increased vIFC compared to normal controls (FDR α < 0.05/2, corrected p < 0.05/2). The right scatter illustrated the significant negative correlations of vIFC with MoCA in the right middle temporal gyrus (as represented by the red rings on the cortical mantle).

**Figure 3:**
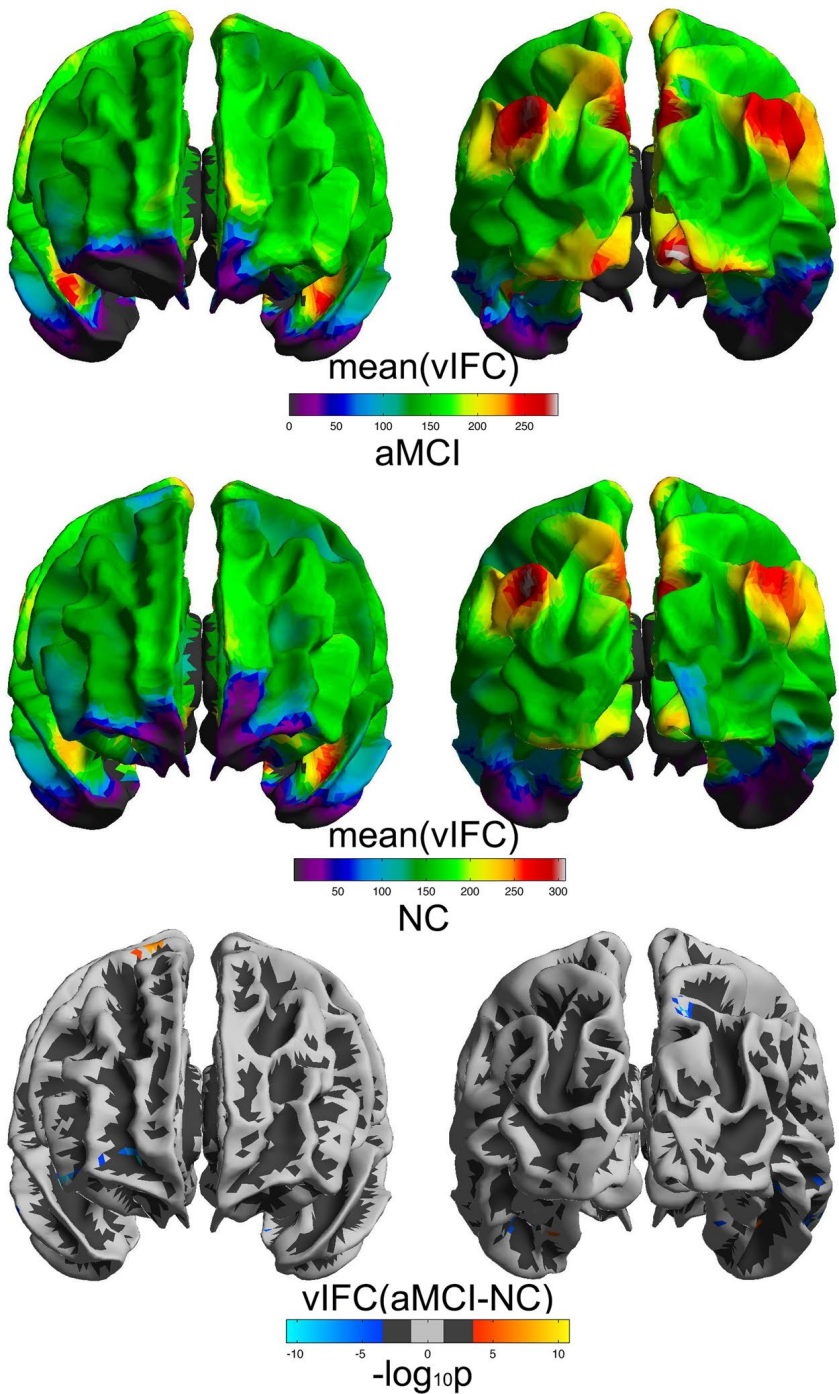
Significant aMCI-NC differences in vIFC across cortical mantle. The cold and warm colors respectively stand for significant decreased and increased vIFC compared to normal controls (FDR α < 0.05/2, corrected p < 0.05/2).

As shown in Figs 2, 3 and S1 (Supplementary Information), SCD, aMCI, AD and NC group exhibited similar vIFC patterns across the entire cortical mantle. We observed significant patient-normal vIFC differences for SCD and aMCI. As shown in Fig. 2, SCD exhibited significant increased vIFC in the left G_and_S_frontomargin, the left G_and_S_transv_frontopol, the left G_oc-temp_med-Parahip and the right G_temporal_middle, as well as significant decreased vIFC in the left G_tempoal_inf and the left S_temporal_inf compared to normal controls. Meanwhile, Fig. 3 showed that aMCI exhibited more widely distributed alterations in vIFC: increased vIFC mainly in the left G_oc-temp_med-Parahip and the right G_precentral, as well as decreased vIFC mainly in the left S_collat_transv_ant, the right G_and_S_frontomargin, the right G_front_inf-Orbital, the right G_orbital, the right S_temporal_inf and the right G_parietal_sup compared to normal control group. Please see Table 1 for a full list of brain regions with significant patient-normal differences. Furthermore, we observed significant vIFC differences between aMCI and all the other three groups, SCD, AD, NC (see Supplementary Information for details).

**Table 1:** Full list of brain regions with significant vIFC differences for aMCI-NC and SCD-NC.

	Contrast	Brain regions	Max (−log_10_p)	VtxMax	Size (mm^2^)	TalX	TalY	TalZ	NVtxs
lh	aMCI < NC	S_collat_transv_ant	−6.437	5622	33.78	−40.6	−25.6	−16.9	4
		G_oc-temp_lat-fusifor	−5.775	10077	8.68	−38	−29.8	−19.3	1
		G_oc-temp_med-Parahip	−4.866	1346	5.09	−21	−15.9	−21.7	1
		S_collat_transv_ant	−4.653	3315	9.44	−43.8	−17.8	−23.2	1
	aMCI > NC	G_oc-temp_med-Parahip	5.519	5487	7.06	−29.5	−21.7	−22.5	1
	SCD < NC	G_temporal_inf	−8.406	7097	25.15	−56.5	−48	−11.7	2
		S_temporal_inf	−7.071	3302	25.33	−55.4	−42.7	−10.1	2
		G_temporal_inf	−4.786	5585	10.11	−55.9	−51.2	−7.4	1
	SCD > NC	G_oc-temp_med-Parahip	6.374	5487	7.06	−29.5	−21.7	−22.5	1
		G_and_S_frontomargin	5.158	8214	21.74	−33.5	51.9	−4.7	2
		G_and_S_frontomargin	5.091	8217	10.65	−28.7	49.2	−4.5	1
		G_and_S_transv_frontopol	5.018	4107	26.33	−21.2	58.2	0.8	2
rh	aMCI < NC	G_and_S_frontomargin	−10.828	8264	54.86	21.5	51	−5.4	5
		G_front_inf-Orbital	−10.123	6652	40.6	47	30.6	−9.3	4
		G_orbital	−9.879	4857	137.82	38.3	24.1	−10.2	17
		G_front_inf-Orbital	−9.263	4846	53.15	48.3	33.2	−9	4
		S_temporal_inf	−8.741	5751	69.53	52.3	−17	−23.4	6
		G_parietal_sup	−8.513	7767	14.72	13.8	−69	47.6	2
		G_parietal_sup	−7.266	1684	47.68	16.6	−67.5	45.7	5
		S_temporal_inf	−6.688	7195	11.21	55.9	−22	−21.3	1
		G_temp_sup-Lateral	−6.073	2725	11.52	46	9.3	−22.6	1
		S_collat_transv_ant	−5.591	10076	8.72	40.9	−35	−16.6	1
		S_collat_transv_ant	−5.552	5623	17.31	40.4	−29.8	−16.5	2
		G_temporal_middle	−5.437	8033	22.32	61.7	−14	−17.4	2
		G_temporal_middle	−4.546	5646	11.43	59.7	−39	−7.1	1
		G_and_S_cingul-Ant	−4.523	3707	27.57	9.3	28.7	−11.1	3
		G_parietal_sup	−4.28	3166	8.23	14.7	−66.7	51.9	1
		S_oc-temp_lat	−4.072	7999	9.34	45.8	−54.7	−7.3	1
		G_temporal_middle	−3.938	8035	11.84	58.1	−19.8	−17.5	1
		G_oc-temp_med-Parahip	−3.686	1367	8.24	26.5	−24.5	−20.7	1
		G_precuneus	−3.592	8363	6.35	11	−68.3	50.7	1
		G_front_middle	−3.509	8216	14.82	35.3	50.1	−4.2	1
	aMCI > NC	G_precentral	7.607	3370	17.47	18.6	−13.7	64.9	3
		G_precentral	5.878	2781	11.09	13.6	−19.2	67	2
		S_collat_transv_ant	4.979	5555	16.22	40.1	−14.3	−20.9	2
		G_precentral	4.543	5767	7.16	22.1	−12.2	62.8	1
	SCD > NC	G_temporal_middle	5.271	2760	10.99	60.5	−30	−12.9	1
		G_temporal_middle	5.151	5740	7.27	53.3	4.5	−25.7	1

AD, Alzheimer’s Disease; aMCI, amnestic Mild cognitive impairment; SCD, subjective cognitive decline; NC, cognitively normal subjects; lh, left hemisphere; rh, right hemisphere.

### Clinical behavioral correlations

As shown in Fig. 2, vIFC in the right G_temporal_middle significantly negatively correlated with total score of MoCA in SCD (p = 6.88e-4, r = −0.39, Bonferroni correction for multiple clusters p < 0.05/9). No other significant correlations were observed.

### Comparison with STD, Corr(in) and Corr(out)

**Figure 4:**
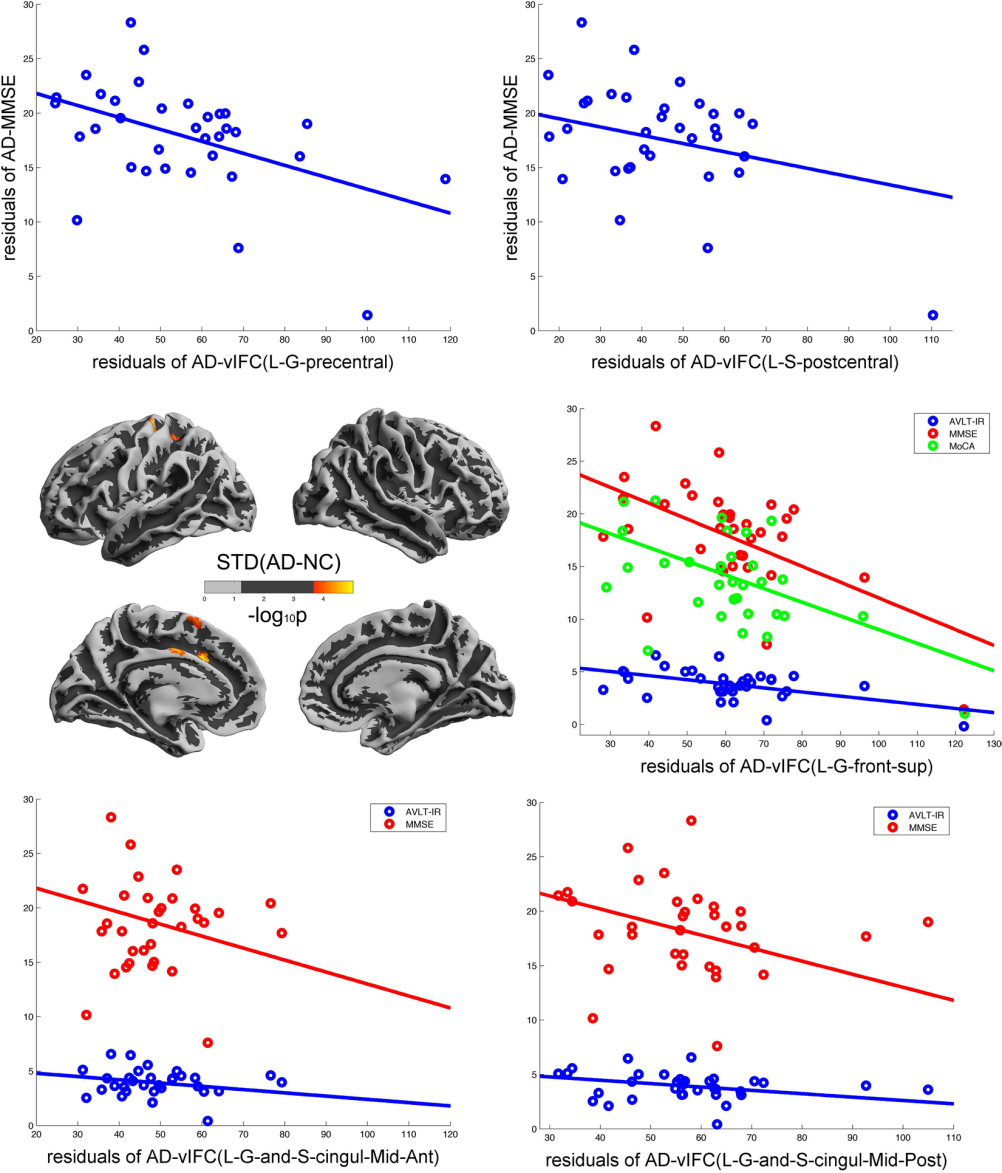
Significant AD-NC differences in STD across cortical mantle. The warm color stand for significant increased STD compared to normal controls (FDR α < 0.05/2, corrected p < 0.05/2). The five scatters represent the significant negative correlations of STD with behavioral measurements.

vIFC exhibited significant patient-normal differences for SCD and aMCI. In comparison, we also performed the same statistics using the three components of vIFC, For Corr(in) and Corr(out), we didn’t find any significant differences. For STD, we found significant increased STD in the left G_precentral, the left G_and_S_cingul-Mid, the left S_oc-temp_lat, the left G_front_sup and the left S_postcentral in AD compared with normal controls (Fig. 4). Also we found some significant negative correlations of STD with behavioral measurements in AD patients: STD in the L-G_precentral versus MMSE (R = −0.4735, P = 0.0071); STD in the L-G_and_S_cingul-Mid-Ant versus AVLT-IR (R = −0.5143, P = 0.0031) and MMSE (R = −0.5312, P = 0.0021); STD in the the left G_and_S_cingul-Mid-Post versus AVLT-IR (R = −0.4705, P = 0.0076) and MMSE (R = −0.4948, P = 0.0047); STD in the L-G_front_sup versus AVLT-IR (R = −0.5085, P = 0.0035), MMSE (R = −0.5588, P = 0.0011) and MoCA (R = −0.5542, P = 0.0022); STD in the L-S_postcentral versus AVLT-IR (R = −0.4699, P = 0.0076). We used Bonferroni correction for multiple clusters p < 0.05/6.

## Discussion

Based on critical transition theory of nonlinear dynamics in physics, here we proposed a vertex-wise Index of Functional Criticality of human brain. This new algorithm employed fMRI time series to calculate the probability of existence of critical transitions for a given vertex. Without knowing connection details of human brain network, the method supplies a great opportunity to characterize and study functional criticality of human brain dynamical system. Also our results showed that the vertex-wise Index of Functional Criticality was rather sensitive compared with the three components of vIFC, STD, Corr(in) and Corr(out). Furthermore, the widely distributed vIFC impairments in aMCI may indicate aMCI as a critical transition across AD progression. Different from previous fMRI methodologies, our vIFC method has a solid theoretical basis and could serve as a neuroimaging marker for future human brain studies from the dynamical perspective.

### Functional Criticality of human brain at millimeter level

Human brain is one of the most complicated systems and could be studied from different spatial scales such as neurons, vertices/millimeters and brain regions. However, the systematic organizational principles should be similar with other biological system and even advanced technology physical system^13^. In more detail, human brain could be modeled using a set of differential equations and then the dynamical characteristics of human brain could be derived. But the construction of the complicated differential equations needs a lot of assumptions^14^. Another commonly used method is just employing the statistical dependence of fMRI time series to construct human brain network^15^. And this is how the studies using fMRI methodology usually do. vIFC just combined the above two kinds of study regimes and have its very unique advantages: there was no need to hypothesize or estimate the connection details of human brain; such kind of ‘black box’ operation could easily be widely applied in large scale clinical or healthy populations; this was the first index to characterize functional criticality of human brain from the spatial scale of millimeters. On the other hand, there was evidence showing that human brain benefits a lot from criticality: the dynamical range, information transmission and information capacity were all optimized at criticality^16^. Therefore, studies on functional criticality of human brain is rather meaningful, and our vertex-wise functional criticality would shed more lights on the understanding of human brain organizational principles from millimeter level in future.

### Characterize dynamical progression of AD: a sensitive neuroimaging marker

The progression of Alzheimer’s Disease (AD) usually comprise three stages, subjective cognitive decline (SCD), mild cognitive impairment (MCI), and dementia due to AD^2^. Although Jack *et al.*^5^ proposed a hypothetical dynamical biomarker model of pathophysiological process in AD, quantitatively characterizing dynamical trajectory of AD progression from the perspective of brain function is still on the very beginning. Here we tried to use patient-normal differences to probe whether there was a critical transition across AD progression. Firstly, for SCD, only few studies reported its functional abnormalities^17–19^. However, our vIFC method detected significant increased and decreased multiple vIFC clusters compared to normal controls. This indicated that vIFC was a rather sensitive method for characterizing brain abnormalities. Secondly, aMCI patients exhibited more patient-normal vIFC differences compared to SCD and AD patients. This indicated that aMCI is a rather special stage, and might be a critical transition across AD progression. Thirdly, in comparison with degree centrality, using vIFC we observed more significant patient-normal differences. Finally, consistent with previous studies^20,21^, the temporal lobe displayed a major damage during AD progression in this study. All these results validated our data analysis, confirmed vIFC as a sensitive neuroimaging marker for future studies, and characterized aMCI as a critical transition across AD progression.

One would expect high average correlation outside the spatial neighborhood for the regions of wide-spread networks such as default-mode or frontal-parietal networks. Therefore, functionally, these networks would have larger vIFC scores. Since in AD particularly DMN connectivity is impaired, for a spatial cluster vIFC score of the voxels associated to DMN will appear increased (due to the denominator in equation 1), although functionally it is expected to decrease (due to the numerator in equation 1). We didn’t find any significant differences between AD patients and normal controls, a likely explanation might be that the diffusion of the activity is impaired during the course of AD due to degeneration, but vIFC score is masked in AD group by the reductions in whole-brain correlations.

### Correlations between MoCA and functional criticality

MoCA was a rapid screening instrument for cognitive dysfunction. It assesses different cognitive domains including attention, executive function and memory. It has been proved with high sensitivity and moderate specificity^22,23^. Also there were few studies, which showed significant correlations of brain structural and functional alterations in SCD with cognitive impairment^3,24^. Here we employed a new algorithm, vIFC to explore the associations of functional criticality of human brain with cognitive impairment. We observed significant negative correlations of vIFC with MoCA total score in the right middle temporal gyrus. Selnes *et al*.^25^ reported impaired brain structures in the middle temporal cortices in SCD. Such overlapping in the middle temporal cortex may underlie associations of brain structure with function. Also, the correlations further validated our data analysis and may promote MoCA as an efficient screening tool for the early warning of SCD diagnosis.

### Limitations

The original DNB method is applied to a data that represents the dynamics of interest of a particular individual. Because of disease complexities and personal variations (e.g., genetic or epigenetic factors), each individual may progress to the same disease through different underlying networks, which hampers the discovery of effective molecule- or model-based biomarkers. So addressing individual variations is critical because it is very difficult to justify if the observed alterations reflect a shift in criticality or any other systematic alterations that are not related to the dynamics. Our study involved four different clinically groups of distinct individuals. Ideally our vIFC method should be first justified in healthy control subjects or by using appropriate randomization techniques. Indeed, we tried to use healthy children’s brain images to do vIFC analysis, but unfortunately we didn’t find any meaningful results. In future we would try to use appropriate randomization technique to further verify our method.

## Conclusions

We proposed a vertex-wise method to describe the possibility of existence of critical transition in human brain, vIFC, based on critical transition theory of nonlinear dynamics. vIFC successfully detect significant patient-normal differences for SCD and aMCI, as well as significant correlates of vIFC in the right middle temporal gyrus with MoCA scores in SCD. However, among the three components of vIFC, only STD detected significant differences between AD patients and NC. Therefore, vIFC could serve as a sensitive neuroimaging marker for future studies; considering much more vIFC impairments in aMCI compared to SCD and AD, our study indicated aMCI as a transitional or critical stage across AD progression.

## Methods

### Participants

Subjects with SCD (n = 47), aMCI (n = 93) and AD (n = 48) were recruited from the memory clinic of the Neurology Department, Xuanwu Hospital, Capital Medical University, Beijing, China. Normal Controls (NC, n = 92) were recruited from local community by advertisements. All the patients were diagnosed by the consensus of two consultant psychiatrists according to the criteria for SCD, aMCI and AD^26–28^. An international effort to establish common standards for SCD, SCD-I^2,29–31^ has been proposed, which is a broad definition of SCD along with specific features that increase the likelihood of preclinical AD in the affected individuals (referred to as SCD plus). In this study all we used were SCD plus. However, for simplicity we still used ‘SCD’ in our manuscript. A standard clinical evaluation protocol, which included Mini-Mental State Exam (MMSE), Auditory Verbal Learning Test (AVLT), Montreal Cognitive Assessment (MoCA), and Clinic Dementia Rating Scale (CDR) evaluated all the subjects. All the subjects had no history of other major neuropsychiatric illness, head injury, alcohol and drug abuse. The medical research ethics committee and institutional review board of Xuanwu Hospital approved this study, and written informed consent was obtained from individual participant prior to data acquisition. The methods were carried out in accordance with the approved guidelines.

### MRI Imaging

All the MRI images were collected on a 3.0 T Siemens scanner (Erlangen, Germany) at Xuanwu Hospital, Capital Medical University. All the participants completed a T1-weighted structural MRI scan with a MPRAGE sequence (TR = 1900ms; TE = 2.2ms; TI = 900ms; FA = 9**°**; matrix = 256 × 256; slice thickness = 1.0 mm; 176 sagittal slices, no gap) and a 478-second resting state fMRI scan using an EPI sequence (TR = 2000ms; TE = 40ms; FA = 90°; number of slices = 28; slice thickness = 4 mm; gap = 1 mm; voxel size = 4 mm × 4 mm × 4 mm; and matrix = 64 × 64). Participants were asked to lie quietly in the scanner with their eyes closed during data acquisition.

### Imaging Data Preprocessing

All of the images were preprocessed using the Connectome Computation System (CCS) platform^32^ with cortical surface reconstruction as a key component. Detailed descriptions of the system can be found in our previous publications^33,34^. Preprocessing comprised structural image preprocessing and functional image preprocessing. The structural image preprocessing was mainly cortical surface reconstruction^35,36^, which included MR intensity inhomogeneity correction, brain extraction, automated segmentation of the cerebrospinal fluid (CSF), white matter (WM) and deep gray matter (GM), production of the GM-WM (white surface) and GM-CSF interface (pial surface), and spatial registration via matching of the cortical folding patterns across subjects. The functional image preprocessing involved the following steps: drop the first 4 EPI volumes to allow for signal equilibration, detect and reduce outliers (spikes), slice timing correction, alignment of each volume to a ‘base’ volume (the mean EPI) to correct for between-head movements, normalization of the 4D global mean intensity into 10000 to allow for inter-subject comparison, regressing out the WM/CSF mean time series and the Friston-24 motion time series^37,38^, filtering the residual time series with a band-pass (0.01–0.1 Hz) filter, removal of both linear and quadratic trends, and alignment of the individual motion-corrected functional image to the anatomical image with a GM-WM boundary-based registration algorithm^39^. Finally, individual preprocessed 4D rfMRI time series were projected onto the *fsaverage5* standard cortical surface with 10,242 vertices per hemisphere and an average spacing of 4 mm^40^.

### Quality Control Procedure

Quality control is very important for solid data analysis. CCS provides a set of complicated quality control procedures: (1) brain extraction or skull stripping, (2) brain tissue segmentation, (3) pial and white surface reconstruction, (4) boundary-based functional image registration, and (5) head motion correction during rFMRI. For those images that the brain extraction was not good, we used two types of thresholds for the FSL BET combined with the FreeSurfer automated skull strip to supply templates for manual editing. Quantitative controls of boundary-based functional image registration (mcBBR <= 0.5) and head motion (maxTran <= 2 mm, maxRot <= 2°, mean FD < 0.2 mm) were also used. Subjects without demographic data like years of education and age were all excluded. And then we selected age-, sex-, and years of education- matched normal controls from NC group. Finally we have 42 SCD (43.1–80.0 years, 16 males), 67 aMCI (44.2–85.1 years, 30 males) and 34 AD (51.9–86.8 years, 12 males) and 54 NC (58.1–83.1 years, 21 males). The detailed participant information was summarized in Table 2.

**Table 2:** Demographic information and behavioral measurements for AD, aMCI, SCD and NC.

	AD (34)	aMCI (67)	SCD (42)	NC (54)	F/ Chi-square values	P values
Age (Years)	51.90–86.80 (69.86 ± 9.00)	44.2–85.1 (66.08 ± 9.35)	43.1–80.0 (66.01 ± 8.0)	58.10–83.10 (67.22 ± 6.15)	1.861	0.138
Gender (M/F)	12/22	30/37	16/26	21/33	1.040	0.792
Education (Years)	0–25 (10.29 ± 4.79)	0–21 (10.73 ± 4.15)	2–18 (11.88 ± 4.17)	0–22 (11.37 ± 5.22)	0.962	0.412
MMSE	3–29 (17.88 ± 5.24)	17–30 (24.97 ± 3.18)	22–30 (27.62 ± 1.85)	20–30 (28.31 ± 2.26)	83.552	0.000
AVLT Immediate Recall	0–6.67 (3.80 ± 1.44)	2.67–10 (5.89 ± 1.62)	4.67–13.67 (7.95 ± 1.86)	6–13.67 (9.17 ± 1.75)	84.931	0.000
AVLT Delayed Recall	0–5 (1.12 ± 1.67)	0–11 (3.82 ± 2.74)	3–15 (8.29 ± 2.69)	4–15 (10.06 ± 2.59)	116.171	0.000
AVLT Recognition	0–12 (4.41 ± 3.02)	0–14 (7.67 ± 3.61)	2–15 (10.81 ± 2.67)	1–15 (11.89 ± 3.02)	46.677	0.000
MoCa	3–22 (13.58 ± 4.84)	13–26 (20.17 ± 3.71)	21–30 (25.91 ± 1.91)	18–30 (26.32 ± 2.84)	100.599	0.000
MoCa Delayed Recall	0–1 (0.13 ± 0.34)	0–5 (0.93 ± 1.18)	0–5 (3.11 ± 1.41)	0–6 (3.18 ± 1.59)	47.705	0.000
MoCa Orientation	0–6 (2.70 ± 1.77)	1–6 (4.98 ± 1.34)	2–6 (5.83 ± 0.75)	5–6 (5.95 ± 0.23)	51.821	0.000

AD, Alzheimer’s Disease; aMCI, amnestic Mild cognitive impairment; SCD, subjective cognitive decline; NC, cognitively normal subjects; MMSE, Mini-mental state examination; AVLT, Auditory verbal learning test; MoCA, Montreal cognitive assessment.

Differences among the samples were tested with ANOVAs (p < 0.05) with LSD or Dunnett’s T3 post hoc comparisons.

### Data availability

The datasets used and generated in this study are available from the corresponding author on reasonable request.

### vIFC Method

Whenever a dynamical system approaches a critical state immediately before phase transition, a subnetwork or a dominant group of variables in the system appears and satisfies the following three conditions^7–9^:

1. The average Pearson’s correlation coefficients across time (PCCs) for the variables within the group drastically increase in absolute value (see *PCC*_*in*_ in Eqn. (1)).
2. The average PCCs of the variables within this group with any other variables (i.e., those outside the group) drastically decrease in absolute value (see *PCC*_*out*_ in Eqn. (1)).
3. The average standard deviations (SD) of the variables in the group drastically increase (see *STD(i*) in Eqn. (1)).

These three conditions demonstrate that appearance of a group of variables with high correlations and strong fluctuations implies the emergence of a critical transition. The special group of variables was called DNB. Based on these three dynamical characteristics of DNB nodes (variables), we proposed a vertex-wise Index of Functional Criticality (vIFC) method for human brain rfMRI datasets. Also in this algorithm, we adopted local information transfer hypothesis (Spatial neighborhoods were likely involved in the same dominant group of vertices), which was inherited from the method of local functional homogeneity (ReHo). The detailed mathematical formula was as following:

1 $${\rm{vIFC}}(i)=\frac{STD(i)PC{C}_{in}}{PC{C}_{out}}=\frac{\sqrt{{\sum }_{n=1}^{N}{({x}_{i}({t}_{n})- < {x}_{i}({t}_{n}) > )}^{2}}\cdot  < PC{C}_{ij}(j\in I) > }{ < PC{C}_{ik}(k\notin I) > }$$

**Figure 5:**
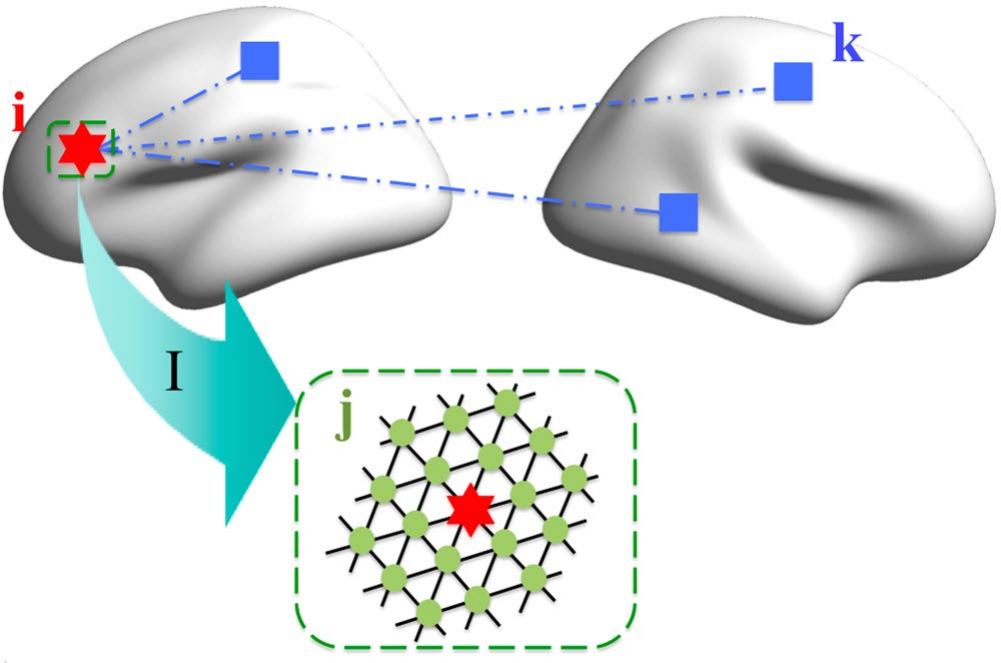
Schematic of the vIFC algorithm across cerebral cortex. There are three types of vertices, i.e., {i}, {j ∈ I}, and {k ∉ I}. Given vertex *i*, *I* stand for its second 2 neighborhood cluster, *j* represent the vertices within I, and *k* represent other vertices (outside the neighborhood).

As illustrated in Fig. 5 and Eqn. 1, there are three types of vertices, i.e., {i}, {j ∈ I}, and {kj ∉ I}. For given vertex *i*, *I* stands for its second-order neighborhood cluster, *j* represents vertices within *I*, and *k* represents other vertices outside the neighborhood. *N* is the number of fMRI BOLD time points. *x*_*i*_*(t*_*n*_) stands for the fMRI BOLD value of the vertex *i* at time *t*_*n*_; PCC stands for the inter-vertex Pearson correlation coefficient across time; STD stands for the standard deviation of the BOLD time series; and < > in the ‘STD’ means averaging across all the time points, < > in the ‘PCC’ means averaging across vertices. Intuitively, based on the three DNB conditions, whenever a system approaches critical states, *vIFC(i)* in the dominant group drastically increases, and thus the variables with high vIFC scores can signal the emergence of critical transition. In another word, vIFC quantitatively measures the possibility of the emergence of critical transitions. The calculation was repeated for each vertex, and we then obtained a vIFC map on the fsaverage5 surface of each participant. All individual vIFC maps were spatially smoothed with a Gaussian kernel with a 10-mm FWHM on *fsaverage5*.

### Three components of vIFC

As shown in the vIFC formula, there were three components that contributed to vIFC values, standard deviation of BOLD time series (STD), within cluster correlation (Corr(in)) and outside cluster correlation (Corr(out)). We also calculated the three components respectively for each participant on fsaverage5 surface, and a Gaussian kernel with a 10-mm FWHM was also performed.

### Statistics

Similar with our previous studies^33,34,41^, we employed FreeSurfer Group Descriptor (FSGD) files to generate a general linear model that considered age, sex, and years of education as covariates with DODS (Different Offset and Different Slope). Finally, the vertex-wise significance values for each contrast of group comparisons were corrected with FDR (False Discovery Rate) method (FDR α = 0.05/2, corrected *p* = 0.05/2). After acquiring the cluster with significant vIFC differences, we also calculated the partial correlations of average vIFC within the cluster and behavioral measurements (MMSE, AVLT and MoCA) after controlling age, sex, and years of education.

## Electronic supplementary material

Supplementary Information
