# Supplementary material for: Impaired Functional Criticality of Human Brain during Alzheimer’s Disease Progression
Source: Sci Rep. 2018 Jan 22;8:1324. doi: 10.1038/s41598-018-19674-7 (PMC5778032; doi:10.1038/s41598-018-19674-7)

Supplementary Information

Impaired Functional Criticality of Human Brain during Alzheimer’s Disease Progression

Lili Jiang^1,2,3*^, Danyang Sui^1,2,4^, Kaini Qiao^1,2,4^, Hao-Ming Dong^1,2,4^, Luonan Chen^5^, Ying Han^6,7,8,9,10*^

^1^CAS Key Laboratory of Behavioral Science, Institute of Psychology, Beijing 100101, China; ^2^Lifespan Connectomics and Behavior Team, Institute of Psychology, Chinese Academy of Sciences, Beijing 100101, China; ^3^Princeton Neuroscience Institute, Princeton University, Princeton, NJ 08544, USA; ^4^Department of Psychology, University of Chinese Academy of Sciences, Shijingshan, Beijing 100049, China. ^5^Key Laboratory of Systems Biology, Innovation Center for Cell Signaling Network, Institute of Biochemistry and Cell Biology, Shanghai Institutes for Biological Sciences Chinese Academy of Sciences, 320 Yue Yang Road, Shanghai 200031, China; ^6^Department of Neurology, XuanWu Hospital of Capital Medical University, Beijing, China, 100053; ^7^Center of Alzheimer’s Disease, Beijing; Institute for Brain Disorders, Beijing, China, 100053; ^8^Beijing Institute of Geriatrics, Beijing, China, 100053; ^9^National Clinical Research Center for Geriatric Disorders, Beijing, China, 100053; ^10^PKU Care Rehabilitation Hospital, Beijing, China, 100053

***Corresponding Author:** Lili Jiang, Institute of Psychology, Chinese Academy of Sciences, No. 16 Lincui Road, Chaoyang District, Southern Building, Room 705, Beijing, 100101, China. Tel: +8610 64864261; Email: [jiangll@psych.ac.cn](mailto:jiangll@psych.ac.cn); Ying Han, Department of Neurology, XuanWu Hospital of Capital Medical University, Beijing, 100053, China. Email: 13621011941@163.com

**Table S1.** Full list of brain regions with significant vIFC differences in SCD-aMCI, SCD-AD and aMCI-AD.

|  | **Contrast** | **Brain regions** | **Max**  **(-log_10_p)** | **VtxMax** | **Size**  **(mm^2^)** | **TalX** | **TalY** | **TalZ** | **NVtxs** |
| --- | --- | --- | --- | --- | --- | --- | --- | --- | --- |
| lh | AD>aMCI | G_oc-temp_med-Parahip | 6.529 | 1346 | 12.85 | -21 | -15.9 | -21.7 | 2 |
|  |  | S_collat_transv_ant | 6.246 | 3315 | 9.44 | -43.8 | -17.8 | -23.2 | 1 |
|  | aMCI<SCD | S_temporal_inf | -6.939 | 5535 | 51.46 | -47.3 | -12.6 | -26.5 | 4 |
|  |  | S_circular_insula_ant | -6.545 | 9848 | 7.63 | -25.2 | 14.8 | -16.2 | 1 |
|  |  | G_orbital | -6.488 | 3249 | 10.73 | -30.1 | 18.5 | -19.5 | 2 |
|  |  | G_temp_sup-Lateral | -6.045 | 2468 | 45.99 | -45.4 | 5.4 | -27 | 3 |
|  |  | G_subcallosal | -4.757 | 2207 | 5.8 | -6 | 19.4 | -8.1 | 1 |
|  | aMCI>SCD | G_temporal_inf | 6.083 | 7097 | 13.21 | -56.5 | -48 | -11.7 | 1 |
| rh | AD>aMCI | G_orbital | 10.263 | 1133 | 37.46 | 40.7 | 45.1 | -7.1 | 3 |
|  |  | G_temporal_middle | 6.798 | 5740 | 7.27 | 53.3 | 4.5 | -25.7 | 1 |
|  |  | G_temporal_inf | 6.504 | 7144 | 23.94 | 56.3 | -54.7 | -9.6 | 2 |
|  |  | Pole_temporal | 5.724 | 2729 | 12.37 | 46.9 | 5.6 | -26.2 | 1 |
|  |  | S_circular_insula_ant | 5.054 | 2723 | 5.19 | 26.4 | 18.5 | -10.9 | 1 |
|  |  | S_oc-temp_lat | 4.97 | 1417 | 7.9 | 45.7 | -35.6 | -15 | 1 |
|  |  | S_circular_insula_ant | 4.967 | 9131 | 7.06 | 29.8 | 22.4 | -12.6 | 1 |
|  |  | G_and_S_frontomargin | 4.716 | 8264 | 10.32 | 21.5 | 51 | -5.4 | 1 |
|  |  | S_collat_transv_ant | 4.46 | 10076 | 8.72 | 40.9 | -35 | -16.6 | 1 |
|  |  | G_front_inf-Orbital | 4.357 | 1136 | 20 | 43.5 | 30.1 | -7.7 | 2 |
|  |  | G_parietal_sup | 4.28 | 1684 | 8.7 | 16.6 | -67.5 | 45.7 | 1 |
|  |  | G_temp_sup-Lateral | 4.107 | 2725 | 11.52 | 46 | 9.3 | -22.6 | 1 |
|  | aMCI<SCD | G_temporal_middle | -13.157 | 5740 | 52.8 | 53.3 | 4.5 | -25.7 | 6 |
|  |  | G_and_S_frontomargin | -12.073 | 8264 | 149.67 | 21.5 | 51 | -5.4 | 13 |
|  |  | G_parietal_sup | -9.707 | 7767 | 14.72 | 13.8 | -69 | 47.6 | 2 |
|  |  | G_front_inf-Orbital | -9.638 | 4846 | 72.74 | 48.3 | 33.2 | -9 | 6 |
|  |  | G_temp_sup-Lateral | -9.488 | 2725 | 44.94 | 46 | 9.3 | -22.6 | 4 |
|  |  | G_front_inf-Orbital | -9.224 | 6652 | 29.65 | 47 | 30.6 | -9.3 | 3 |
|  |  | Pole_temporal | -8.959 | 2729 | 12.37 | 46.9 | 5.6 | -26.2 | 1 |
|  |  | G_parietal_sup | -8.226 | 1684 | 36.35 | 16.6 | -67.5 | 45.7 | 4 |
|  |  | S_temporal_inf | -7.808 | 6121 | 44.89 | 56.4 | -33 | -12.7 | 4 |
|  |  | G_orbital | -7.261 | 4857 | 44.14 | 38.3 | 24.1 | -10.2 | 5 |
|  |  | G_oc-temp_med-Parahip | -6.401 | 1367 | 24.13 | 26.5 | -24.5 | -20.7 | 3 |
|  |  | S_circular_insula_ant | -6.047 | 6659 | 10.21 | 36.6 | 27.2 | -7.3 | 1 |
|  |  | S_circular_insula_ant | -6.014 | 2723 | 10.53 | 26.4 | 18.5 | -10.9 | 2 |
|  |  | G_and_S_cingul-Ant | -5.728 | 3707 | 27.57 | 9.3 | 28.7 | -11.1 | 3 |
|  |  | S_oc-temp_lat | -5.713 | 7999 | 38.05 | 45.8 | -54.7 | -7.3 | 4 |
|  |  | G_temporal_middle | -4.753 | 5646 | 11.43 | 59.7 | -39 | -7.1 | 1 |
|  |  | G_temporal_inf | -4.685 | 3328 | 13.81 | 49.9 | -55.5 | -7.8 | 1 |
|  |  | G_and_S_cingul-Ant | -4.354 | 5967 | 27.23 | 4.9 | 24.8 | -8.7 | 4 |
|  |  | G_temporal_middle | -3.7 | 161 | 7.01 | 61.8 | -8.7 | -18.3 | 1 |
|  |  | S_temporal_inf | -3.664 | 10131 | 13.35 | 56.2 | -42.6 | -9.1 | 1 |
|  |  | G_oc-temp_med-Parahip | -3.63 | 3285 | 4.92 | 22.4 | -23.6 | -18.2 | 1 |
|  | aMCI>SCD | Pole_occipital | 7.66 | 10002 | 45.88 | 19.3 | -97.1 | -0.4 | 3 |
|  |  | S_collat_transv_ant | 6.331 | 9996 | 8.44 | 40.3 | -22.8 | -17.6 | 1 |
|  |  | G_and_S_paracentral | 4.971 | 7489 | 9.12 | 9.1 | -30.3 | 71.2 | 2 |
|  |  | S_collat_transv_ant | 4.85 | 5555 | 16.22 | 40.1 | -14.3 | -20.9 | 2 |
|  |  | Pole_occipital | 4.675 | 1374 | 22.52 | 15.2 | -97.6 | 2.3 | 2 |
|  |  | G_precentral | 4.655 | 862 | 10.79 | 11.5 | -20.3 | 67.7 | 2 |
|  |  | G_precentral | 4.28 | 2772 | 6.59 | 11.8 | -15.3 | 66.7 | 1 |

**Figure S1.** vIFC maps of AD and NC.


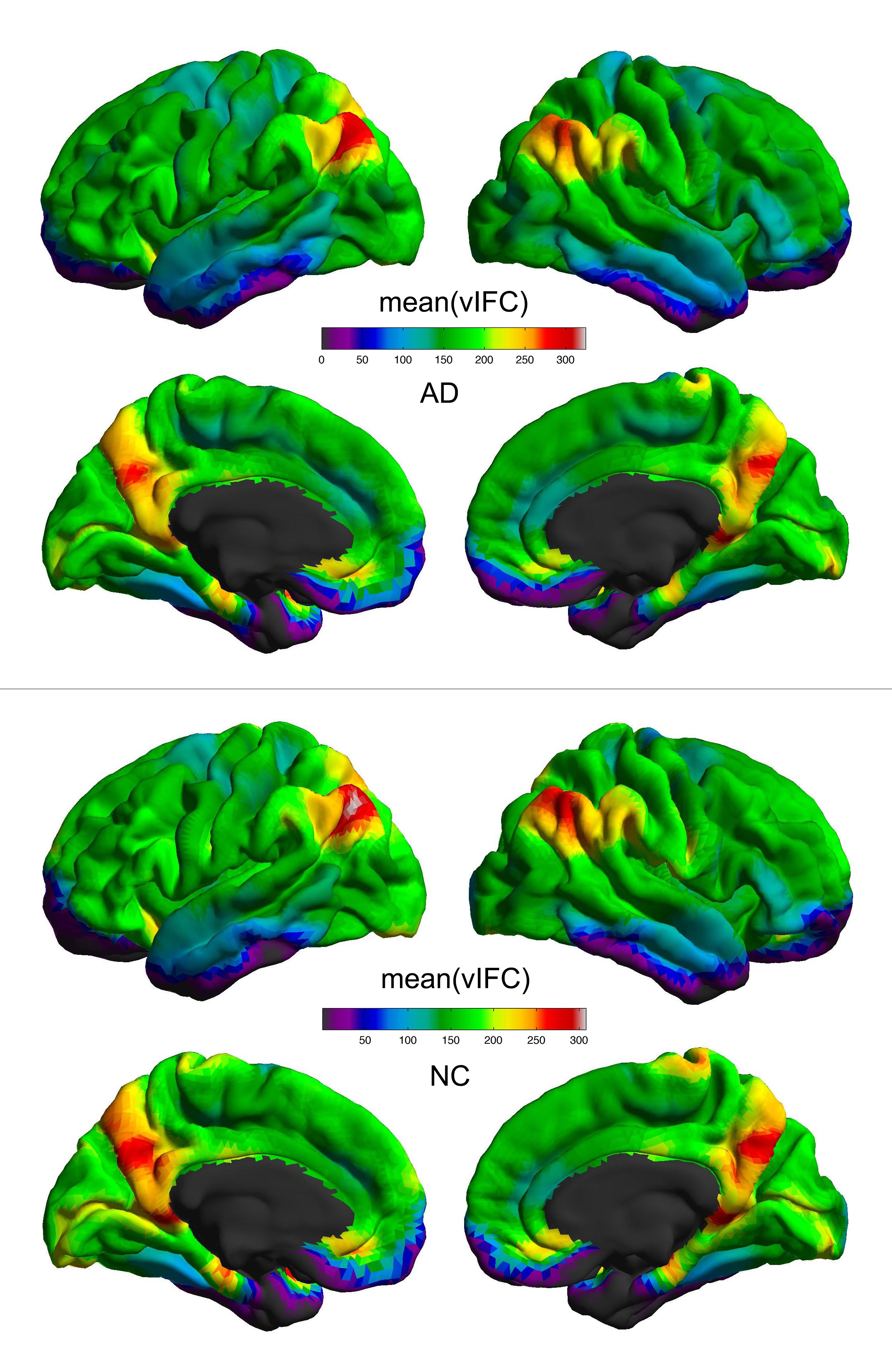

Supplement: Supplementary file 1 — Supplementary Information [file 41598_2018_19674_MOESM1_ESM.doc]
